# Impact of cytokine release on ventricular function after hepatic reperfusion: a prospective observational echocardiographic study with tissue Doppler imaging

**DOI:** 10.1186/s12871-015-0080-2

**Published:** 2015-07-25

**Authors:** Marco P. Zalunardo, Martin Schläpfer, Beatrice Beck-Schimmer, Burkhardt Seifert, Donat R. Spahn, Dominique Bettex

**Affiliations:** 1 Institute of Anaesthesiology, University and University Hospital Zürich, Raemistrasse 100, 8091 Zürich, Switzerland; 2 Institute of Physiology, University Zurich Irchel, 8057 Zurich, Switzerland; 3 Department of Anesthesiologoy, University of Illinois at Chicago, 60612 Chicago, USA; 4 Department of Biostatistics, Epidemiology, Biostatistics and Prevention Institute, University of Zurich, 8001 Zurich, Switzerland; 5 Institute of Anaesthesiology, University Children’s Hospital Zurich, 8032 Zurich, Switzerland

**Keywords:** Liver transplantation, Cardiac function, Postreperfusion syndrome

## Abstract

**Background:**

Postreperfusion syndrome and haemodynamic instability are predictors for poor outcome after liver transplantation. Cytokine release has been claimed to be responsible for postreperfusion syndrome. However, the underlying pathophysiologic mechanism is not clarified.

The aim of this prospective observational study was to correlate cardiac performance (measured by transoesophageal echocardiography (TEE), Doppler and Tissue Doppler Imaging (TDI)) to plasmatic cytokines: IL-6, IL-8, CXCL1, TGF-β and CD40L at 5 different time points during liver transplantation.

**Methods:**

Seventeen consecutive patients scheduled for orthotopic liver transplantation, age 18 to 75 years without contraindication for transoesophageal echocardiography were included. Patients were monitored with TEE and TDI.

Systolic and diastolic cardiac function, MAP, MPAP, CVP, PCWP, CO and blood samples for cytokine assays were recorded or collected after induction, 15 min after vena cava inferior clamping, 2 to 5 min after reperfusion, 60 min after reperfusion and at the end of surgery.

**Results:**

Mean arterial pressure and catecholamine requirements remained unchanged, MPAP, CVP and CO increased, SVR decreased after unclamping. Postreperfusion syndrome did not develop. The haemodynamic parameters and the variations of TEE parameters were consistent with the volume load changes during clamping and declamping and did not reveal systolic or diastolic cardiac dysfunction. All cytokines, except TGF-β, increased.

**Conclusion:**

These findings suggest, that significant cytokine release during liver transplantation is not necessarily coincident with haemodynamic instability and impaired cardiac function.

**Trial registration:**

ClinicalTrials.gov: NCT00547924

## Background

Reperfusion of the liver graft is one of the most critical periods during liver transplantation. Several factors may contribute to immediate haemodynamic instability after reperfusion, so called the postreperfusion syndrome (PRS). Free radicals, proinflammatory cytokines, metabolic acidosis, electrolyte disturbances and elevation of cyclic guanosine monophosphate (cGMP) have been referred to as possible causative factors [1–3]. Haemodynamic instability in the context of PRS is defined as a decrease of mean arterial blood pressure of more than 30 % in the first 5 min after graft reperfusion continuing for at least 1 min [4, 5]. Its incidence is reported to be 8-50 % and it is associated with substantial increase in perioperative complications and lower early survival [6–8]. Several studies identified cardiac failure as primary cause for PRS [9, 10]. However, both the responsible pathophysiologic mechanism for haemodynamic instability after reperfusion and the significant correlation between cytokine release and impaired cardiac function have not been demonstrated yet.

The purpose of this study was to measure proinflammatory cytokines and systolic and diastolic cardiac function with TEE using 2D, Doppler and Tissue Doppler Imaging (TDI) at different stages before and after hepatic reperfusion. Doppler echocardiography in association with TDI is a very sensitive method for the evaluation of diastolic function [11] and has not been applied in liver transplantation so far. We hypothesized, that an increase of washed in proinflammatory mediators from the liver graft might impair ventricular function after reperfusion.

## Methods

### Ethics

This prospective, observational study was approved by the local Ethics Committee (Kantonale Ethikkommission Zürich, approved on 4^th^ May 2006) Ref.Nr. StV 11-2006, ClinicalTrials.gov Identifier: NCT00547924.

### Patients

Twenty consecutive patients on the Swiss Transplant liver transplantation waiting list were enrolled between 03/2007 and 02/2010. Written consent of all patients was obtained prior to transplantation. Patients between 18 to 75 years were included. Exclusion criteria were participation in a different study, contraindications for transoesophageal echocardiography: constrictions, tumours, varicose veins or a diverticulum in the oesophagus and cervical instability.

### Measurement time points

Mean arterial pressure (MAP), mean pulmonary arterial pressure (MPAP), central venous pressure (CVP), pulmonary capillary wedge pressure (PCWP), cardiac output (CO), systemic vascular resistance (SVR) and all blood samples for cytokine assays and arterial blood gas analysis were recorded or collected at the following time points: baseline, after induction of anaesthesia (A), 15 min after vena cava inferior clamping (prior to reperfusion) (B), 2 to 5 min after reperfusion and declamping of vena cava inferior (C), 60 min after reperfusion (D), at the end of surgery (E). TEE measurements were performed at time points A, B, C and D.

### Anaesthesia and monitoring

Anaesthesia and monitoring were performed following an institutional protocol. Anaesthesia was induced with propofol, fentanyl and rocuronium or atracurium and maintained with sevoflurane or isoflurane, fentanyl and atracurium. A central venous line, a pulmonary artery catheter, a femoral arterial and one or more peripheral venous lines were installed. TEE was performed, unless there was a recent bleeding from oesophageal varicose veins or hypocoagulability with prothrombin time (Quick value) less than 20 % (institutional laboratory reference) or platelet count less than 30000/ml. Other indications and contraindications referred to the practice guidelines for perioperative TEE of the American Society of Anesthesiologists [12, 13]. All echocardiographic studies were performed by the same experienced cardiac anaesthetist (Dominique Bettex), who is an expert in TEE.

### TEE measurements and technique

Data acquisition was performed by a multiplane transoesophageal echocardiography probe and an echocardiography apparatus GE Vivid 7 or Vivid Q (System V7 or VQ, GE Vingmed, Horten, Norway, 5.5 MHz transducer). Measurements with TEE were collected at the four time points A, B, C, and D to assess systolic and diastolic function of the left ventricle (LV): fractional area change (FAC) in transgastric short-axis view (enddiastolic area – endsystolic area/enddiastolic area), ejection fraction (EF) using the modified Simpson’s rule [14] in mid-oesophageal four- and two-chamber views or Teichholz method [15] in transgastric short-axis view were recorded for the assessment of LV systolic function. Different measurements of systolic function were used, because the transgastric views were not always available, particularly during liver grafting. In case of a discrepancy of the various measurements we relied on EF measured according to Simpson’s rules. Color M-mode Doppler was performed in mid-oesophageal four-chamber view in the standard manner defined previously to obtain the flow propagation velocity (Vp), indicative of the LV relaxation [16]. The transmitral Doppler flow was obtained with the Doppler interrogation window placed at the leaflet tips of the mitral valve and the maximum transmitral velocities of the early filling or E wave and late filling or A wave, the ratio E/A, and deceleration time (DT) of the E wave were measured. The isovolumetric relaxation time (IVRT) was obtained after a slight modification of the interrogation window into the left ventricular outflow tract. Finally, tissue pulse-Doppler imaging was obtained during apnoea on the mid-oesophageal four-chamber view, placing the region of interest at the level of the mitral annulus on the lateral as well as on the medial aspects; the maximum velocities of systolic wave (S’), and diastolic waves (E’ and A’) were collected and the ratios E’/A’ and E/E’ were calculated. The diastolic function was assessed as described previously [11, 17]. The ratio E/E’ was used as criteria for high LV filling pressure as follow: E/E’ > 15, LAP > 18mmHg; E/E’ < 8, LAP = 10-12mmHg [18, 19].

### Enzyme-linked immunosorbent assay (ELISA)

Concentrations of various cytokines in plasma were determined as previously described [20]: interleukin-6 (IL-6), interleukin-8 (IL-8), chemokine (C-X-C motif) ligand 1 (CXCL1), transforming growth factor-β (TGF-β), CD40 ligand (CD40L) and tumour necrosis factor alpha (TNF-α). Sandwich ELISAs were performed according to the manufacturer’s protocol (R&D Systems Europe Ltd, Abingdon, UK). Detection limits of the ELISA were determined by the manufacturer (R&D Systems Europe Ltd) as follows: for IL-6 9.37 pg/ml, for IL-8, TGF-β and CXCL1 31.25 pg/ml and for CD40L 15.65 pg/ml.

### Statistics

Continuous variables are presented as mean with standard deviation (SD) and are compared between different time points using the paired *t*-test. Variables were logarithmised for these analyses if appropriate. *P*-values less than 0.05 are considered significant. The Bonferroni-correction was used to address the problem of multiple comparisons. As there are 5 comparisons between time points A/B, A/C, B/C, C/D, and D/E for each variable, *p*-values less than 0.01 are considered significant in these analyses. IBM SPSS Statistics 20 (SPSS Inc., Chicago, IL) was used for statistical analyses.

## Results

After enrolment 3 patients had to be excluded for different reasons: pancreatic carcinoma of the donor, diagnosed in situ (n = 1), unavailability of the TEE specialist (n = 1) and blood samples for cytokine assays were not cooled and centrifuged after sampling (n = 1). Patient characteristics and surgical data of the remaining 17 patients are shown in Table 1.

**Table 1:** Patient characteristics and surgical data

Age, years (n = 17)	59^1^ (42-68)
Female, n	5
Male, n	12
Duration of Surgery	440^2^ (100)
MELD score	15.7^2^ (9.9)
Alcoholic Cirrhosis, n	3
Cryptogenic Cirrhosis, n	3
Hepatitis C Cirrhosis, n	3
Hepatitis B Cirrhosis, n	2
Hepatitis C and Hepatocellular Carcinoma, n	2
Hemochromatosis, Hepatocellular Carcinoma, n	1
Hepatocellular Carcinoma, n	1
Graft Rejection, n	1
Primary Biliary Cirrhosis. n	1
LV EF <50 %, n	0
Coronary Heart Disease, n	1
0-1 cardiovascular risk-factor^3^, n	10
≥2 cardiovascular risk-factors^3^, n	7

^1^Median (range)

^2^Mean (standard deviation)

^3^Modifiable cardiovascular risk factors according to WHO: Hypertension, smoking, diabetes and overweight and obesity; physical inactivity was excluded (Mendis S, Puska P, Norrving B: Global Atlas on Cardiovascular Disease Prevention and Control, 2011, ISBN-13 9789241564373)

### Surgical procedures

Standard technique with cross clamping of the vena cava inferior was used in 11 patients, partial clamping of the vena cava inferior (piggyback technique) in 6 cases. One re-transplantation and 3 living donor transplantations were performed. Veno-venous bypass was not used. Blood flush of 200-400 ml was drained from the liver graft prior to reperfusion according to the institutional protocol. In all cases surgery was performed by one of two experienced liver surgeons.

### Donor and graft criteria

Detailed information about donor age, the cause of death or the reason for donation and cold ischemia time can be found in Table 2.

**Table 2:** Donor and Graft Criteria

Age in years		57.4 (19.5)
Diagnosis	Intracranial haemorrhage	10
	Living donation	3
	Cerebral oedema due to meningeoma	1
	Trauma	1
	Anoxia after myocardial infarction	1
	Asphyxia	1
Cold ischemia time (minutes)		440 (216)

### Haemodynamic parameters

**Fig. 1:**
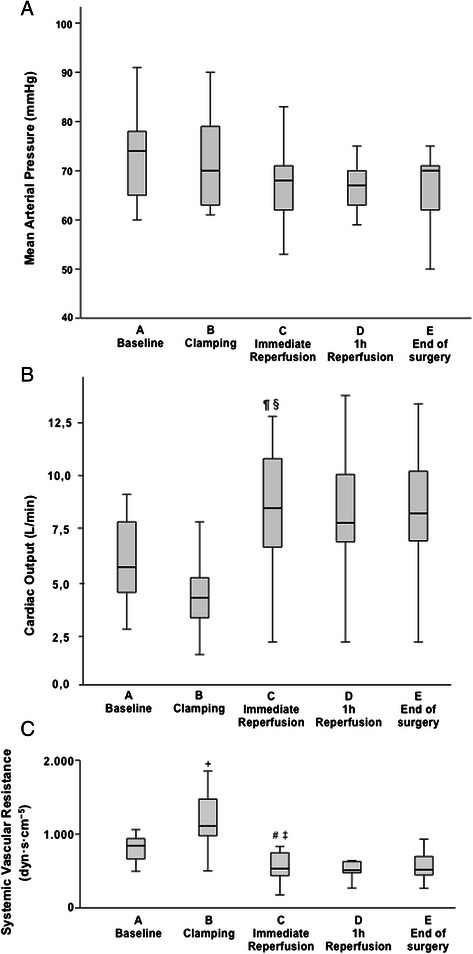
Haemodynamic parameters. Mean arterial pressure **(a)**, cardiac output **(b)** and systemic vascular resistance **(c)** at the different time points of the study. ¶ increase of cardiac output from **a** baseline to **c** immediate reperfusion (*p* < 0,001). § increase of cardiac output from **b** clamping the vena cava inferior to **c** immediate reperfusion (*p* < 0,001). + increase of systemic vascular resistance from **a** baseline to **b** clamping the vena cava inferior (*p* = 0,004). # decrease of systemic vascular resistance from **a** baseline to **c** immediate reperfusion (*p* = 0,002). ‡ decrease of systemic vascular resistance from **b** clamping the vena cava inferior to **c** immediate reperfusion (*p* < 0,001)

Haemodynamic parameters were compared regarding changes from baseline to clamping (A to B), baseline to immediate reperfusion (A to C), clamping to immediate reperfusion (B to C), immediate reperfusion to 1 h after reperfusion and 1 h after reperfusion to end of surgery. Results are shown in Tables 3, 4 and Fig. 1. Significant changes in haemodynamic parameters were documented from cross clamping to immediate reperfusion for all parameters except for stable MAP: MPAP, CVP and CO increased, whereas SVR decreased. Haemodynamic criteria for PRS were not fulfilled.

**Table 3:** Haemodynamic parameters

	A	B	C	D	E
	Baseline	Clamping	Imm. Reperfusi on^1^	1h Reperfusion	End of surgery
MAP (mmHg)	73 (9)	71 (9)	66 (12)	66 (5)	67 (7)
MPAP (mmHg)	22 (6)	17 (7)	25 (7)	23 (8)	23 (7)
PCWP (mmHg)	15 (5)	12 (6)	18 (7)	14 (6)	16 (6)
CVP (mmHg)	13 (4)	9 (6)	14 (5)	12 (6)	12 (5)
CO (L/min)	5.8 (1.9)	4.4 (1.8)	8.5 (2.8)	8.1 (2.9)	8.1 (2.9)
SVR (dyn · s · cm^−5^)	900 (325)	1298 (768)	680 (642)	672 (430)	652 (464)

^1^Immediate reperfusion

**Table 4:** Changes of cytokines, echocardiographic and haemodynamic parameters

Cytokines^4^				Echocardiographic Parameters				Haemodynamic Parameters			
IL 6	A/B	↑	*p* < 0,001	DT			n.s.	MAP			n.s.
	B/C	↑	*p* = 0,007	IVRT			n.s.				
	A/C	↑	*p* < 0,001	FP vel	A/C	↑	*p* = 0,009	MPAP	B/C	↓	*p* < 0,001
IL 8	A/B	↑	*p* = 0,01		B/C	↑	*p* = 0,001				
	A/C	↑	*p* = 0,001	E lat	A/B	↓	*p* < 0,001	CVP	B/C	↑	*p* = 0,001
	C/D	↑	*p* = 0,003		B/C	↑	*p* < 0,001				
CXCL1	A/C	↑	*p* < 0,001	A lat	A/C	↑	*p* = 0,001	PCWP	B/C	↑	*p* < 0,001
	B/C	↑	*p* = 0,006	E/A lat	A/B	↓	*p* = 0,003				
TGF-β	n.s.			S’ lat			n.s.		B/C	↑	*p* < 0,001
CD40L	n.s.,	↑	trend for A/C	S’ sept			n.s.				
				E sept	A/B	↓	*p* = 0,002	SVR^4^	A/B	↑	*p* = 0,004
					B/C	↑	*p* < 0,001		A/C	↓	*p* = 0,002
				A sept	A/C	↑	*p* = 0,007		B/C	↓	*p* < 0,001
				E/A sept	A/B	↓	*p* = 0,004				
				E/E sept			n.s.				
				EDA			n.s.				
				ESA	C/D	↑	*p* = 0,005				
				EF			n.s.				
				Peak E	A/B	↓	*p* < 0,001				
					B/C	↑	*p* < 0,001				
				Peak A	B/C	↑	*p* = 0,002				
				E/A	A/B	↓	*p* < 0,001				
					B/C	↑	*p* = 0,001				

^4^Values are logarithmized

n.s. = not significant

### Vasopressors

The continuous noradrenaline administration after clamping was 9.4 ± 8.6 μg/min and hence similar to the continuously administered dose after reperfusion 11.0 ± 7.0 μg/min (B to C). In only 6 out of 17 patients a small bolus of noradrenaline, adrenaline or neosynephrine was required.

### TEE parameters

Transoesophageal echocardiography was uneventful in all patients, there was no oesophageal bleeding after insertion of the echo probe. Several measurements of systolic and diastolic function were performed simultaneously to the collection of the haemodynamic parameters. The results are shown in Tables 4 and 5. Statistically significant variations of the transmitral flow (E,A, E/A), flow propagation velocity (FPvel) and early and late peak diastolic velocity (E’, A’, E’/A’) of the mitral annulus were documented, mostly between baseline (A) and after clamping of the vena cava (B), as well as immediately after reperfusion of the liver (C). These variations were compatible with acute intravascular volume variations and did not demonstrate any systolic or diastolic dysfunction at any time during transplantation. There were no statistically significant changes between baseline (A) and post reperfusion steady state (D).

**Table 5:** Echocardiographic Parameters

	A	B	C	D
	Baseline	Clamping	Imm. Reperfusion^1^	1h Reperfusion
DT (msec)	236 (49.3)	227 (43.8)	225 (41.8)	223 (47.7)
IVRT (msec)	89 (29.1)	102 (36.5)	90.5 (34.7)	99.2 (34.9)
FP vel (cm/sec)	54 (16.3)	45 (15.1)	63 (16.3)	63 (15.8)
E’ lat (cm/sec)	12 (3.5)	9.2 (2.4)	12.4 (2.8)	12.3 (2.6)
A’ lat (cm/sec)	11.3 (3.4)	11.7 (4.1)	14.1 (3.8)	13.4 (3.6)
E’/A’ lat	1.15 (0.5)	0.9 (0.4)	0.9 (0.3)	0.95 (0.3)
E/E’ lat	5.9 (2)	5.2 (1.8)	6.5 (2.7)	7 (2.8)
S’ lat (cm/sec)	9.6 (2)	11.2 (3.5)	12 (4.1)	11.9 (4.4)
S’ sept (cm/sec)	9 (3.1)	8.6 (4)	9.2 (1.8)	9.1 (2.1)
E’ sept (cm/sec)	8.2 (2.7)	5.8 (2.3)	9.7 (3.3)	9.2 (2.4)
A’ sept (cm/sec)	8.2 (2.9)	8.4 (3.2)	10.4 (3.2)	10.3 (2.9)
E’/A’ sept	1.1 (0.4)	0.8 (0.4)	1 (0.3)	0.95 (0.2)
E/E sept	8.9 (3.4)	8.7 (3.4)	8.2 (3.4)	9.35 (3.6)
EDA (cm^2^)	21.4 (6.7)	16.9 (7)	20.5 (5.5)	24 (6.5)
ESA (cm^2^)	10 (4.6)	7.1 (3.6)	6.8 (2.5)	8.3 (2.3)
EF^2^ (%)	72 (9.4)	78 (9)	79 (7.4)	78 (10.9)
Peak E (cm/sec)	67.7 (16.5)	46.1 (13.2)	75 (21.7)	80.2 (14.8)
Peak A (cm/sec)	56.8 (16.3)	48.1 (12.3)	62.4 (17.7)	67.2 (18.3)
E/A	1.27 (0.44)	0.96 (0.32)	1.21 (0.25)	1.23 (0.25)

^1^Immediate reperfusion

^2^EF obtained by Simpson’s rules

### Cytokines

Absolute values are indicated in Table 6, significant changes of values in Table 4. Comparing time points A to D an increase of IL-6, IL-8 was observed, comparing A to C: IL-6, IL-8 and CXCL1 increased. No significant changes were observed for TGF-β and CD40L (Tables 4 and 6). For IL-6 an increased concentration was found at time points B and C compared to A (for both *p* < 0.001). At time point C IL-6 value was higher than at time point B (*p* = 0.007). Interleukin-8 concentrations were higher at time point B as well as C in comparison to A (*p* = 0.01 and *p* = 0.001, respectively). At time point D concentration significantly exceeded the value from time point C (*p* = 0.003). A significant change was measured for CXCL1 at time point C compared to A (*p* < 0.001) as well as to B (*p* = 0.006). Concentrations of TGF-β and CD40L did not differ over time. TNF-α was below detection range.

**Table 6:** Cytokines

	A	B	C	D	E
	Baseline	Clamping	Imm. Reperfusion^1^	1h Reperfusion	End of surgery
IL-6	30 (103)	216 (145)	313 (203)	547 (451)	552 (499)
IL-8	26 (52)	42 (60)	49 (70)	123 (184)	132 (109)
CXCL1	89 (259)	104 (277)	173 (330)	166 (321)	145 (278)
TGF-β	93 (78)	104 (86)	83 (57)	98 (63)	100 (63)
CD40L	1344 (4711)	1174 (4033)	1493 (4798)	1290 (4468)	1284 (4462)

^1^Immediate reperfusion

## Discussion

In contrast to our hypothesis, we found no postreperfusion syndrome and cardiac performance was not impaired in terms of systolic or diastolic function despite increased cytokine levels throughout the course of liver transplantation. Moreover, haemodynamic and echocardiographic parameters reflected the physiological haemodynamic changes due to clamping and unclamping of the vena cava and the consecutive measures like volume loading or administration of catecholamines.

Haemodynamic instability after reperfusion of the liver graft is a major concern in liver transplantation and correlated to poor outcome [6]. In the last two decades many causal factors have been investigated, but none of them has proven the primarily responsible. Even the amount of catecholamines used for liver transplantation was analysed, but no significant correlation with cytokine release was found [21]. The aim of the present study was to evaluate cardiac performance during liver transplantation and the reperfusion period via TEE and to assess the simultaneous cytokine release. We found decreased filling pressures, cardiac output and a compensatory increase of SVR after clamping. The reverse was documented after unclamping. The significant decrease of SVR after unclamping and reperfusion may be interpreted as a cytokine induced effect, also seen after cardiopulmonary bypass [22]. MAP was maintained and CO increased in terms of a compensatory hyperdynamic state. Transoesophageal echocardiographic parameters proceeded similarly. There was no sign of systolic or diastolic dysfunction at any time during the transplantation. However, we are aware of the fact that FAC or EF rather reflect the adaptation to the loading conditions of the heart than inotropy. However, s’, being is less load dependent than FAC or EF, was relatively stable over the course of the transplantation and confirmed our conclusion. Unfortunately, we did not keep track of the acceleration time of s’, which might have been a less load dependent echocardiographic assessment of systolic function. The variations in transmitral flow, flow propagation velocity and early and late peak diastolic velocity of the mitral annulus were all compatible with the relevant volume variations during the clamping of the vena cava and immediately after the reperfusion of the liver. They varied according to the haemodynamic parameters and recovered at the end of the procedure.

Two cytokines are considered major cardiac depressants: IL-6 [23] and TNF-α [24] and therefore especially interesting to answer the question raised in this study. TNF-α was below the detection level in all of our patients.

In comparison to former liver transplantation trials we also have assessed markers that act on the various different inflammatory cells (neutrophils, macrophages, T-cells) and expanded our search pattern of inflammatory mediators to determine also molecules, not having been evaluated before. Although not significant for all parameters, there was an increase in cytokine levels during liver transplantation.

Most recently, Bezinover and collegues suggested an association between plasma cyclic guanosine monophosphate levels and haemodynamic instability during liver transplantation [3]. Similar to their previous study [21] they compared again the amount of catecholamines used to the levels of cGMP. However, choosing the amount of catecholamines as an indicator of haemodynamic stability may be misleading. Many other factors may contribute to catecholamine use during liver transplantation: patients often have a more or less pronounced hyperdynamic circulation with splanchnic vasodilation and a collateral circulation [25] advanced cirrhosis may lead to cardiomyopathy [26]. In addition the recipient may suffer from cardiovascular comorbidities. The preoperative coagulation profile may influence the amount and pace of blood loss, which might also impact on the haemodynamic situation, and catecholamine use of the recipient. Also the skills and experience of the surgeon, which have a proven impact on outcome in advanced bariatric surgery [27], the hospital’s standard operating procedures for haemodynamic management and the individual experience and skills of the anaesthetist, might all impact on the use of catecholamines in liver transplant patients. The results of this study have to be interpreted in the background of the low caseload and all factors mentioned above. Furthermore, the considerably varying occurrence of PRS in the literature underlines this statement. The incidence reported ranges from 8 % to 50 % [6, 8]. However, at our institution we have a detailed protocol for the anaesthetic management of liver transplantation and a specially trained team of consultants for liver and lung transplantation. In addition, one or the other of only two surgical team leaders was involved in all cases. This may have counteracted to individual differences and possibly strengthened the relevance of our results.

As mentioned above, there was no PRS and no impaired cardiac function during the whole procedure, while the level of most proinflammartory cytokines increased significantly (with exception of TNF-α, TGF-β and CD40L). The increase was similar to earlier studies, where either coincidental haemodynamic impairment was found [28] or not present [21]. Increased cytokines without impact on cardiac function are also regularly seen after cardiopulmonary bypass in cardiac surgery [22].

A possible explanation for not observing PRS in our study population might be a rather low MELD-score, a good preoperative cardiac function as well as the good quality of donor organs. Cardiac output seems to adapt pretty well in response to stress in patients with a low MELD score, but even at this early stage of the disease, heart mass and the enddiastolic volume increase with higher scores [29]. As the disease progresses, the changes in structure and function aggravate [30]. In most patients, diastolic dysfunction seems to progress along with the progress of the disease [31], whereas systolic dysfunction only develops in a subset of patients [32].

Study limitations might be, that our patients had a rather low MELD score and a good cardiac baseline function. This makes the study population more homogenous on one hand but is likely not to reflect the full spectrum of changes related to end stage liver disease, especially in patients with a high MELD score. Also the low patient number may be considered a limitation of the current study. Strength of the study is the quality of the data and detailed echocardiography data. Since only our most experienced echocardiologist performed all echoes and measurements, we can assume that the quality of the measurements is very high and an inter-investigator variation can be excluded. The quality of the grafts was considered very good and although we observed a significant increase of some cytokines. We cannot exclude that a more pronounced cytokine release could have impacted on cardiac function.

We hope that our study lays ground for a larger study, observing patients in a more advanced stage of end stage liver disease, as PRS may be more frequent in this patient population and may thus uncover whether the pathophysiology of PRS is related to cardiac function or to vasoplegia.

## Conclusion

In conclusion, echocardiographic and haemodynamic findings did not show neither impaired systolic and diastolic cardiac function nor severe haemodynamic instability during reperfusion of the liver graft, although there was an increase in cytokine levels during liver transplantation. These findings suggest, that significant cytokine release during liver transplantation is not necessarily coincident with haemodynamic instability and impaired cardiac function.

## Abbreviations

A wave: Peak late (atrial contraction) transmitral flow velocity
A’lat/sept: Late diastolic peak tissue velocity of the lateral and septal mitral annulus using tissue pulsed wave Doppler imaging
CD40L: CD40 ligand, soluble CD40
cGMP: Cyclic Guanosine Monophosphate
CO: Cardiac Output
CVP: Central Venous Pressure
CXCL1: Chemokine (C-X-C motif) ligand 1
DT: Deceleration Time of the E Wave
E/A: Ratio of the early over late transmitral flow velocities
EDA: Enddiastolic Area
E/E’: Ratio of the early transmitral flow over the early diastolic peak velocity of the mitral annulus
EF: Ejection Fraction
E’lat/sept: Early diastolic peak tissue velocity of the lateral and septal mitral annulus using tissue pulsed wave Doppler imaging
ESA: Endsystolic Area
E wave: Peak early transmitral flow velocity
FAC: Fractional Area Change
FP vel: color M-mode flow propagation velocity
IL-6: Interleukin-6
IL-8: Interleukin-8
IVRT: Isovolumetric Relaxation Time
LV: Left Ventricle
MAP: Mean Arterial Pressure
MELD: Model of End-stage Liver Disease
MPAP: Mean Pulmonary Arterial Pressure
PCWP: Pulmonary Capillary Wedge Pressure
PRS: Post Reperfusion Syndrome
S’lat/sept: Peak systolic velocity of the lateral and septal mitral annulus using tissue pulsed wave Doppler imaging
SVR: Systemic Vascular Resistance
TDI: Tissue Doppler Imaging
TEE: Transoesophageal Echocardiography
TGF-β: Transforming Growth Factor-beta
TNF-α: Tumour Necrosis Factor-alpha
2D: Two-dimensional echocardiography
